# Exonic enhancers: proceed with caution in exome and genome sequencing studies

**DOI:** 10.1186/s13073-016-0277-0

**Published:** 2016-02-08

**Authors:** Nadav Ahituv

**Affiliations:** Department of Bioengineering and Therapeutic Sciences, University of California San Francisco, San Francisco, CA 94158 USA; Institute for Human Genetics, University of California San Francisco, San Francisco, CA 94158 USA

## Abstract

Exonic enhancers (eExons) are coding exons that also function as enhancers of the gene in which they reside or (a) nearby gene(s). Mutations that affect the enhancer activity of these eExons have been associated with human disease. Therefore, eExon mutations should be taken into account in exome and genome sequencing projects, not only because of the ability of these mutations to modify the encoded proteins but also because of their effects on enhancer activity.

## Exonic enhancers

Exonic enhancers (eExons) are protein-coding exons that have an additional function as enhancers — gene regulatory elements that instruct promoters as to when, where and at what levels they should be active. Enhancers are activated by the binding of transcription factors and cofactors, which subsequently leads to the activation of their target promoters, either through looping interactions between the enhancer and the promoter or via other mechanisms such as tracking or chromatin modifications [1]. eExons have been shown to regulate the gene in which they reside [2, 3] or even (a) neighboring gene(s) [4].

eExons were discovered by carrying out functional gene regulatory assays. In an enhancer assay the potential enhancer sequence — in this case the coding exon — is placed in front of a minimal promoter (a promoter that should only drive expression if it has an enhancer in front of it) followed by a reporter gene, and checked for its ability to turn on the reporter gene. Experiments in which these assays were used showed that eExons were able to drive expression of a reporter gene (see [2] for an example). eExons can also be discovered using comparative genomics [3, 5, 6]. For example, in a comparison of 29 mammalian genomes, human protein-coding sequences were scanned for regions that have low synonymous substitution rates, which could suggest that they have additional functions, such as being enhancers [6]. This analysis showed that over a quarter of all human protein-coding genes contain these synonymous constraint elements. eExons can also be detected using chromatin immunoprecipitation sequencing (ChIP-seq), DNase I hypersensitive site sequencing (DNase-seq) or other genomic technologies that can identify enhancers in an unbiased manner [4, 7].

**Fig. 1:**
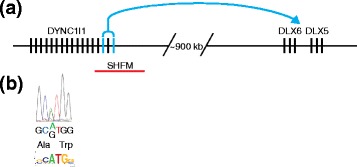
*DYNC1I1* exonic enhancers (*eExons*) regulate *DLX5* and *DLX6*. **a** The *DYNC1I1*-*DLX5*/*6* locus has two known eExons, *DYNC1I1* exons 15 and 17 (colored in *blue*), that are functional limb enhancers and were shown to interact with *DLX5* and *DLX6* [4]. A 106 kb deletion (*red line*) that contains these eExons was found in an individual with split hand and foot malformation (*SHFM*) [10]. **b** A fictional example of a mutation in an eExon that could be overlooked in exome or genome sequencing studies. The chromatogram shows a synonymous mutation in an eExon that could leave the protein sequence unchanged but could affect a transcription factor binding site (logo plot below) leading to changes in the enhancer function of this eExon. *DLX5* Distal-less homeobox 5, *DLX6* Distal-less homeobox 6, *DYNC1I1* Dynein cytoplasmic 1 intermediate chain 1

Mutations in eExons could lead to human disease by altering their enhancer activity. eExons 15 and 17 of the dynein cytoplasmic 1 intermediate chain 1 (*DYNC1I1*) gene are examples of eExons that have been associated with human disease (Fig. 1a). These eExons were shown to be functional enhancers in the developing limb using mouse transgenic enhancer assays. They were also shown to interact with the promoters of distal-less homeobox 5 (*DLX5*) and distal-less homeobox 6 (*DLX6*) in the developing limb [4]. These promoters reside ~900 kb away from *DYNC1I1. DLX5* and *DLX6* are important for limb development and have been associated with split hand and foot malformation (SHFM) in humans [4]. Analysis of patients with SHFM found several chromosomal aberrations that overlap *DYNC1I1* exons 15 and 17 (Fig. 1a) [4, 8, 9], which suggests that alterations in these exons could lead to the SHFM phenotype.

## Coding mutations should be carefully examined

Genomic analyses have also shown that eExons can be quite common in the genome, making up an estimated 7 % of the putative enhancers detected using ChIP-seq [4]. Furthermore, ~15 % of human codons are thought to have sites that are bound by transcription factors (termed duons) on the basis of footprinting analyses of DNase-seq data [7]. Despite being common, the consequences of nucleotide changes on the enhancer function of eExons are usually not taken into account in mutation analyses. Massively parallel reporter assays have shown that the essential functional enhancer sequence of eExons is intertwined with the protein-coding sequence, with both nonsynonymous, synonymous and splice junction mutations having similar deleterious effects on enhancer activity [10]. The transcription factor binding sites were found to be the main constrictive force governing the enhancer function of eExons in this assay. Therefore, a mutation in an eExon, even a synonymous mutation or a mutation in a splice junction, could alter the enhancer activity of this regulatory element and have phenotypic consequences independent of alterations to the protein sequence.

Numerous exome sequencing, whole-genome sequencing and copy number variant (CNV) studies that aim to identify mutations that cause disease or other phenotypic changes have been carried out or are in progress. More than 17 % of single nucleotide variants (SNVs) in coding sequences that overlap a potential functional transcription factor binding site are estimated to alter the site itself [7]. In addition, 13.5 % of coding SNVs that have been associated with disease through genome-wide association studies overlap transcription factor binding sites; 12 % of these SNVs are synonymous and 88 % are nonsynonymous mutations [7]. However, computational analyses in exome or genome sequencing studies are primarily focused on detecting protein-modifying mutations in coding exons and do not specifically consider mutations in eExons that could alter enhancer activity. Therefore, several disease-causing mutations could have been overlooked. For example, a coding mutation in the limb-related *DYNC1I1* eExons in a patient with SHFM would probably be considered non-deleterious and ignored in an exome or genome sequencing study (also due to *DYNC1I1* not having a known role in limb development), unless these sequences were known to function as eExons (Fig. 1b).

## Fixing the problem: how to take eExons into account in mutation analyses

We need to be more conscious of eExons and take them into account when analyzing CNVs and short-sequence variants in exome or genome sequencing data. However, this is not an easy task. Enhancers tend to be cell-type-specific and so eExons could be active only in a specific cell type or tissue, which would make their detection complex. Nevertheless, there are numerous genomic datasets (such as ENCODE or the Roadmap Epigenomics datasets) in which enhancers for various cell types or tissues are annotated, and these datasets will keep on growing. A combined database that provides a list of cell-type-specific or tissue-specific eExons would greatly assist researchers and could be integrated in computational protocols or programs that carry out mutation analyses. The use of programs that predict the effect of regulatory variants on coding sequences, or tools that treat sequences in an unbiased manner regarding their location (that is, in which coding and noncoding mutations are treated similarly), could and should be used to identify changes in eExons that adversely affect their regulatory function.

Another limitation is that an eExon could regulate a nearby gene and not the gene in which it resides, as is the case for the *DYNC1I1* eExons (Fig. 1). Researchers, despite being aware of the presence of eExons, might ignore a variant in a gene that does not have a known function or that does not fit with the phenotype being analyzed. The use of patient gene expression data, such as RNA sequencing (RNA-seq) data, could aid in the identification of a regulatory problem and the gene that is differentially regulated as a consequence. In addition, the use of chromosome conformation datasets [obtained through Hi-C or chromatin interaction analysis by paired-end tag sequencing (ChIA-PET)], when available for the specific cell type or tissue being studied, could assist in assigning target genes to these eExons and these datasets should be taken into account, as should be done when analyzing noncoding enhancers.

In summary, we have not been and are not currently paying sufficient attention in genome and exome sequencing projects to the effects of coding mutations on enhancer activity and other functional elements that could reside in exons. Other than enhancer activity that could reside in exons, these functional elements could include splicing enhancers, RNA secondary structures, microRNA target sites and even dual-coding genes. To conclude, eExons need to be kept in mind when carrying out mutation analyses, in particular for unsolved cases.

## Abbreviations

*DLX5*: Distal-less homeobox 5
*DLX6*: Distal-less homeobox 6
*DYNC1I1*: Dynein cytoplasmic 1 intermediate chain 1
eExon: Exonic enhancer
SHFM: Split hand and foot malformation
